# Leveraging Digital Health to Heal the Planet

**DOI:** 10.1055/s-0046-1816588

**Published:** 2026-11-03

**Authors:** Zerina Lokmic-Tomkins

**Affiliations:** 1 School of Nursing and Midwifery, Monash UniversityClaytonVictoriaAustralia; 2 Health and Climate Initiative, Monash UniversityAustralia

**Keywords:** Climate change, Health informatics, Mitigation, Adaptation, Resilience, Environmental sustainability, Greenwashing

## Abstract

Human-driven climate change is reshaping global and planetary health, with rising temperatures, extreme weather, and environmental degradation affecting all life on Earth. As healthcare and digital technology evolve, digital health has the potential to contribute significantly to the global climate agenda, provided that the principles of environmental sustainability are integrated into daily digital technology developments and operations. Regardless of the political milieu in which we live, environmental sustainability can be strengthened by the ongoing global transition to renewable energy to reduce digital technology’s carbon footprint and by genuinely addressing the environmental impact of digital health interventions at organisational levels. This keynote examines the links between climate change and digital health, and emphasizes the need for proactive systems thinking engagement in greening the digital health sphere. Practical strategies are presented to create greener digital health systems and models of care, covering systemic and individual actions. Harnessing digital health to mitigate, rather than exacerbate, the climate crisis, we, as members of the healthcare sector, take meaningful climate action to protect the health of the people and the planet. By doing so, digital health practitioners pave a sustainable path for healthier planetary futures.

## 1. Introduction

In moments of reflection, it is natural to feel a deep concern for the state of our planet. New records are shattered yearly with reports of the hottest global temperatures, more extreme weather events, and catastrophic floods and bushfires, all amid rising greenhouse gas emissions (GHGs) [ 1 ]. The (un)helpful discussions of Earth’s ‘tipping points’, defined as “critical threshold[s] beyond which a system reorganises, often abruptly and/or irreversibly”, further alarm and possibly paralyse and confuse societal climate discourse by its sense of inevitability [ 2 ]. Urgent calls for climate action are too often met with slow, fragmented responses [ 3 ] or, more recently, with a complete political retreat, as evidenced in the latest United States of America (USA) leadership actions [ 4 ]. This action is against the backdrop of climate scientists warning us for decades about the perils of climate change [ 5 ], and the advancements in science and technology that are profoundly transforming human society, weaving the world into a vast digital network of instant communication, surveillance, and limitless information and potential for climate action.

Digital innovation’s promise lies in its immense potential to support global and planetary health through equitable access to healthcare, education, and global economic opportunities [ 6 – 8 ]. On the other hand, the rapid expansion of digital technology comes at a cost: deepening the digital divide, accelerating environmental destruction, and enabling the spread of misinformation that erodes trust in scientific knowledge [ 6 , 7 ]. Nowhere is this more evident than in the social media discourse surrounding climate change, where disinformation deliberately distorts the human-driven (anthropogenic) causes and consequences of climate change and environmental degradation [ 9 ]. While current geopolitical turmoil threatens to derail global climate action, it is important to remind ourselves that leadership comes in many forms, not just from political leaders [ 10 , 11 ]. While the work will be more challenging because of the loss of USA’s leadership though withdrawal from the Paris Agreement under the United Nations Framework Convention on Climate Change again [ 4 , 12 ], and from the World Health Organization [ 13 ], decisions with enormous geopolitical, health, economic and environmental consequences. However, it is important to remember that another 194 countries, including Brazil, China, European Union, India, Russian Federation and United Kingdom (UK), other major GHG emitters, remain signatories of the Paris Agreement [ 14 ] ( Figure 1 ). Symbolically, UK just released its Nationally Determined Contributions plan committing to 81% emission reduction by 2035 [ 15 ].

**Figure 1:**
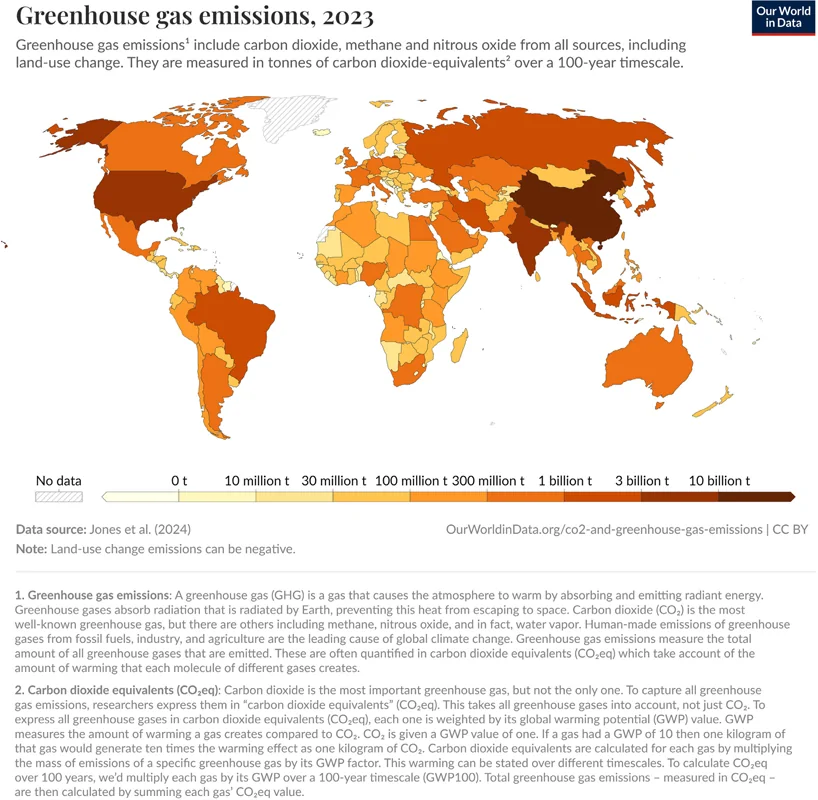
. 2023 annual greenhouse gas emissions, including land use. The figure used under commons licence CC-BY [ 18 ] as per https://ourworldindata.org/grapher/total-ghg-emissions permission

For the digital health community, today is also a critical inflection point for how human-driven digital health systems continue to reduce dependence on fossil fuels, the destruction of the natural world due to extraction of natural resources and global pollution, the three crises facing the planet today [ 16 ]. Our contribution to climate action must embed an environmentally conscious approach to the design, implementation, and scaling of any digital health initiative at all healthcare system levels. The choices we make today on whether to embed environmental sustainability into healthcare’s digital transformation or continue on an unsustainable trajectory, will define the legacy we leave for future generations.

The sustainable pathway forward for digital health is the one that recognizes digital health both as a driver of healthcare innovation and a key stakeholder in environmental stewardship [ 17 ]. Our mission is to harness digital health to mitigate, rather than exacerbate, the climate crisis, to heal rather than harm the planet. So, what actions can we take today, within our shared climate reality, to ensure that future generations inherit a digital healthcare system that heals rather than harms?

## 2. Our Shared Climate Reality

The ongoing release of large amounts of GHGs (carbon dioxide, methane, nitrous oxide) into the atmosphere increases the greenhouse effect, trapping more solar heat and leading to increased global warming ( Figure 2 ) [ 5 ]. Global GHG concentrations are the highest in at least 800,000 years. Specifically, the atmospheric concentration of CO2 (carbon dioxide) has increased from approximately 278 ppm (parts per million) in 1750 to 420 ppm in 2023, an increase of 51%, while global average CH4 (methane) concentrations increased from 729 during preindustrial time to 1934 ppb (parts per billion) in 2023, an increase of 165%, while N2O (nitrous oxide) increased from 270 ppb in 1750 to 336.9 ppb in 2023, a 124% increase [ 1 ]. CO2 remains in the atmosphere for centuries, meaning that CO2 emissions today will impact the climate for many future generations. Methane, a far more potent but shorter-lived gas, lasts around 7 to 12 years but has 80 times the warming potential of CO₂ over a 20-year period [ 19 ]. Nitrous oxide, predominantly generated through agricultural and industrial processes, lasts over a century and contributes to global warming and ozone depletion [ 20 ].

**Figure 2:**
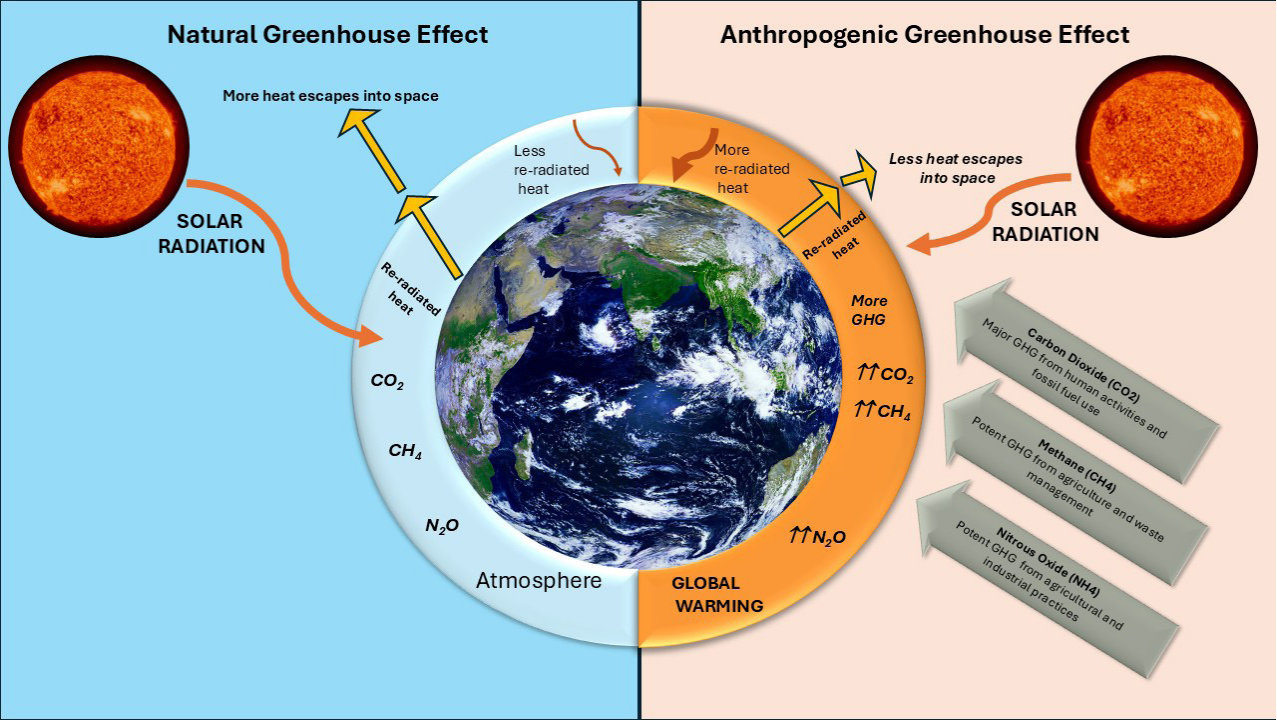
. The natural greenhouse effect regulates Earth’s temperature by trapping some of the sun’s heat. Without it, the planet would be significantly colder. Carbon dioxide (CO2) is an essential GHG for maintaining a stable climate. However, excessive CO2 emissions from human activities intensify heat retention in the atmosphere, driving climate change.

The choices we make today on the emission of GHGs determine the concentration of heat-trapping gases in our atmosphere for a long time. Based on where the GHG emissions originate, they are categorised into Scopes 1, 2, or 3 emissions. According to the Greenhouse Gas Protocol, Scope 1 refers to direct emissions made by an entity—for example, a hospital directly produces the emissions; Scope 2 emissions capture indirect emissions arising from the hospital’s energy purchases; and Scope 3 emissions capture all indirect GHG emissions that result from hospital’s activities such as purchasing wound care supplies [ 21 ]. Identifying and targeting Scopes 1, 2 and 3 emissions within healthcare settings allows entities to understand and mitigate healthcare’s contribution to GHG emissions [ 22 ].

It is unequivocal that the magnitude of human activities, such as burning fossil fuels for power generation and transport, agriculture and burning of agricultural waste, deforestation, and industrial emissions, drive global warming and destabilise the natural balance of the climate systems [ 5 , 23 ]. As the climate warms, the glaciers melt, sea level rises, biodiversity is lost, freshwater availability is threatened, nutrient cycles are disrupted, and more extreme and unpredictable weather patterns that lead to disasters are evident [ 5 , 12 , 24 ]. Extreme bushfire activity in South America has contributed to rising land-use emissions in 2024 [ 25 ], and it is likely that similar can be expected in 2025. The average global temperature in 2024 was 1.6°C above pre-industrial levels since records began in 1850 [ 26 ]. While the initial reaction may be that the world has failed to limit global warming to 1.5°C as per the global Paris Agreement [ 12 ], it is important to remember that the success of the Paris Agreement will be measured against a longer timescale than the past two years as the Intergovernmental Panel on Climate Change (IPCC) defines climate change as: “a change in the state of the climate that can be identified (e.g., by using statistical tests) by changes in the mean and/or the variability of its properties and that persists for an extended period, typically decades or longer” [ 27 ]. Nevertheless, the efforts to urgently and rapidly reduce GHG emissions must continue, as the reality is that global warming will not stop unless net-zero emissions are achieved [ 5 ]. The amount of GHGs emitted over time is approximately proportional to the increase in global temperatures over that same period, meaning that for every tonne of GHGs emitted, the world will see approximately the same amount of global warming [ 5 ]. While it is a simple, scientific fact that humans must stop adding GHGs to the atmosphere by eliminating GHG emissions at their source and finding ways to remove excessive GHGs from the atmosphere to stop global warming [ 5 ], transitioning to GHG-free futures is neither easy nor simple.

## 3. The Climate Cost of Digital Health

The global environmental GHG footprint of healthcare is estimated at 4.4% of global emissions [ 22 ]. Similar to global emissions, healthcare in affluent countries has higher GHG emissions. For example, the US health sector contributes 8.5% of the national GHG emissions and 25% of the global health sector’s GHG emissions [ 28 ]. The UK’s health sector contributes approximately 4% of national emissions [ 29 ], while Australia’s healthcare contributes 7% of the nation’s GHG emissions [ 30 ]. However, these emissions do not specifically report GHG emissions of digital health technologies and their applications in healthcare. The exact contribution of digital health is not known. However, Information and Communication Technology (ICT) devices and services are responsible for 2.1-3.9% of GHG emissions [ 31 ]. Without conscious efforts to implement greener ICTs, this will likely increase to 14% GHG by 2040 due to the growing energy consumption required to make and operate ICT devices [ 32 ].

When thinking about the climate cost of digital health, we need to consider the cost from a planetary perspective, both as direct and indirect impacts on the Earth as a complex system made of five interconnected parts, often described as spheres or subsystems: the Atmosphere, Hydrosphere, Biosphere, Cryosphere, and Geosphere ( Figure 3 ). These subsystems are responsible for the global cycling of energy, water, nutrients and other trace substances [ 33 ]. A complex web of interacting processes connecting these subsystems makes them interdependent on one another as they regulate Earth’s climate system. When thinking about reducing the digital health contribution to climate change, a systems thinking approach is needed that recognises how the Earth’s spheres are impacted by digital health-related activity.

**Figure 3:**
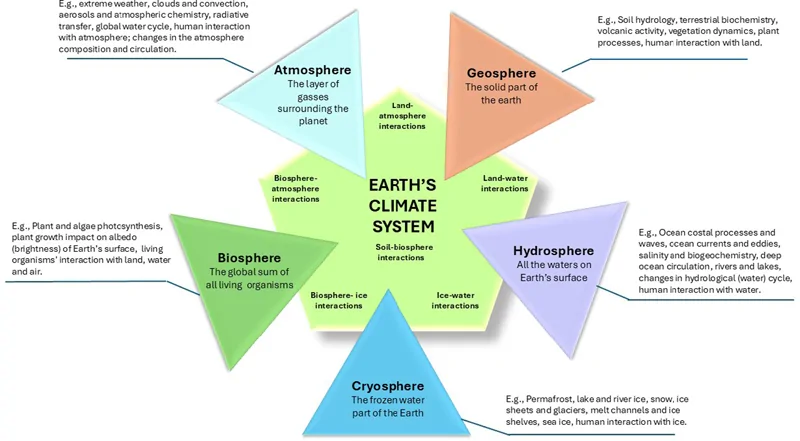
. The climate system consists of interconnected components, including the atmosphere, biosphere (life), cryosphere (ice), hydrosphere (water), and geosphere (land), which are impacted by human influences. These components interact dynamically through various processes, represented by the pentagon as an ongoing process.

### 3.1 The Impact on the Atmosphere

The atmosphere refers to a mixture of gases that surround the planet Earth. Its function is to shield the planet from harmful ultraviolet radiation and to keep the planet warm by trapping health, and maintaining moderate temperatures needed to support life on the planet [ 34 , 35 ]. The anthropogenic contribution of GHGs is polluting the atmosphere and leading to increased trapping of heat in the atmosphere, causing average global temperatures to rise and changes that we know as climate change [ 34 , 35 ]. Air pollution negatively impacts human health across many body systems, and across the lifespan, including pregnancy [ 23 ].

Digital health heavily relies on digital infrastructure such as data centres, network equipment, and personal devices, all of which require energy to use, manufacture, transport, and dispose of digital technologies [ 17 ]. The energy consumed is also directly linked to the vast amount of healthcare data generated, processed and stored. Data centres that require energy to run also require cooling systems, which also require energy to run. When we consider that each clinical encounter, each test, each treatment plan and follow-up management are documented and that this data then needs to be accessible, the energy needed to support these processes is vast. Data centres and data transmission networks alone account for 1 to 1.5% of global electricity demand, with projections suggesting this could rise as artificial intelligence (AI) and big data analytics expand in healthcare and boost demand for data services [ 36 ]. It is estimated that in 2030, the Scope 2 pollutants of US data centres alone could cause approximately 600,000 asthma symptom cases and 1,300 premature deaths, while, depending on the locations, training an AI model of the Llama-3.1 scale could produce an amount of air pollutants equivalent to driving a passenger car for more than 10,000 Los Angeles to New York City round trips [ 37 ]. The push for real-time analytics, precision medicine, and AI-assisted diagnostics means that more energy-intensive models will be deployed, further amplifying these impacts and straining global energy resources.

Another example is telemedicine, often praised for reducing patient travel emissions, which has its own dark side. The infrastructure supporting video consultations, streaming services, cloud storage, and cybersecurity measures requires continuous energy input [ 38 ]. Depending on the network and data centre efficiency, a single hour-long video call emits between 150 to 1,000 grams of CO₂, uses 2-12 litters of water and a land area about the size of an iPad Mini [ 39 ]. Multiplied across millions of consultations worldwide, the cumulative impact of video-based consultations is substantial, especially when video quality demands lead to higher data transfer and energy use.

Beyond energy concerns, the rapid turnover of medical technology fuels an unsustainable cycle of electronic waste (e-waste). The healthcare sector generates an estimated 50 million metric tonnes of e-waste annually, including outdated diagnostic equipment, disposable sensors, and short-lifespan wearables [ 40 ]. Alarmingly, the global e-waste recycling process remains highly inefficient, with only 20% of e-waste being properly recycled [ 40 , 41 ]. Most are shipped to low-resource countries, where unregulated and unsafe recycling practices, including burning plastic to retrieve copper wires, expose workers and their families to harm. This is also tied to climate injustice, as the Western world exports its e-waste to impoverished nations that are among the most affected by climate change yet receive the least support in addressing its impacts [ 7 , 41 ]. Without a transition to renewable energies, the energy needed to support ICTs will continue to come from burning fossil fuels and releasing GHGs into the atmosphere.

### 3.2 The Impact on the Hydrosphere

Globally, there is a decrease in freshwater supply [ 42 ]. Projections indicate that global water demand will rise by 55% between 2000 and 2050, driven by a significant increase in manufacturing (+400%), thermal power generation (+140%), and domestic use (+130%) [ 43 ]. For example, in 2018, Google reported water consumption of 15.8 billion litres, up from 11.4 billion litres in 2017. Similarly, Microsoft reported using 3.6 billion litres in 2018, an increase from 1.9 billion litres in 2017 [ 43 ]. In the context of digital health, which relies heavily on cloud computing and AI, large amounts of cooling water will be required to operate these systems and maintain them at optimal temperatures. This is not helped by continuous increase in healthcare data collection, currently constituting 30% of global data [ 44 ]. Ironically, in 2019 it was estimated that 97% of all global data produced by hospitals each year is unused [ 45 ]. Contamination of freshwater sources is also of concern when e-waste-related hazardous materials such as mercury, lead, and cadmium leach into water, soil and air, creating significant public health risks across the lifespan [ 41 , 46 , 47 ].

### 3.3 The Impact on the Biosphere

The biosphere includes all living organisms on the planet [ 16 ]. These organisms depend on clean air, water and soil. The greatest threat is likely to arise from mining activities for rare minerals and improper disposal of e-waste. For example, high levels of cadmium, found in nickel-cadmium batteries, semiconductors and photoconductive materials [ 48 ], in the biosphere can accumulate in plants and animals and, through the food chain, lead to toxic effects in animals, such as kidney disease and reduced fertility and developmental abnormalities [ 46 ]. Exposure to cadmium leads to reduced plant growth due to interference in photosynthesis and nutrient uptake [ 49 ]. Decreased crop yields threaten food security [ 46 ]. Other contaminants of concern include lead, selenium, antimony and its compounds, plastics containing brominated flame retardants, mercury and mercury compounds and polychlorinated biphenyls, all components of e-waste [ 48 ]. Then, there is the extraction of rare earth metals from the Earth for digital health devices, which leads to deforestation and biodiversity loss through impact on wildlife habitats [ 16 ].

### 3.4 The Impact on the Cryosphere

The cryosphere refers to the frozen components of the Earth’s system, including glaciers, ice caps, sea ice, and permafrost [ 34 ]. The indirect impact of digital health on the cryosphere is due to its contribution to global warming, as discussed above. Global warming accelerates ice melt, leading to increased sea-level rise. The warming consequences are increased risk of tidal flooding, increased salinity in the coastal soil, which impacts produce farming, destruction of plant and animal habitats, and disruption of freshwater supplies [ 5 ]. Loss of ice coverage on the planet also increases the absorption of the Sun’s energy into the ocean and the land [ 5 , 33 ]. In the 2024 State of the Climate Report, the glacier mass loss in 2022/2023 was estimated to correspond to a volume of water discharged by the Amazon River in about one month, or approximately 5 times as much water as there is in the Dead Sea [ 1 ]. The indirect impact of digital health on cryosphere is also due to the mining of rare earth elements that leads to deforestation, contributing to global warming [ 5 , 33 ].

### 3.5 The Impact on Geosphere

The geosphere refers to the Earth’s solid components, including landforms, rocks, minerals, and soil [ 34 ]. The obvious impact is that mining for rare minerals from the Earth leads to habitat destruction, soil degradation, and deforestation where environmentally aware processes are not followed. The more alarming threat is improper recycling of e-waste, which contaminates soil and groundwater by leaching minerals and compounds harmful to plant, animal and human health as described above for the biosphere impact [ 41 , 47 , 48 , 50 ]. This also brings us to the issue of climate injustice [ 51 ], as e-waste is often sent to low-income nations for recycling and disposal, sometimes through informal or unregulated channels [ 52 ]. In many of these countries, waste management and labour costs are low, but working conditions can be challenging, and environmental oversight is often limited [ 47 ]. The incineration of ICT plastics contributes to local air pollution and toxin release, affecting the health of already vulnerable populations [ 52 ]. Where child labour is prevalent, children are exposed to hazardous e-waste chemicals linked to serious health risks, including cancer [ 50 ]. Similarly, many of our institutions are guilty of donating outdated medical equipment and digital health technologies to lower-income countries to support healthcare services. However, when these devices become obsolete, the receiving nations may lack the resources to manage the resulting waste, which may end up in a landfill. This raises important ethical questions: How can we collaborate with colleagues in these countries to develop locally led environmentally sustainable solutions that support livelihoods of local populations? Without sustainable design principles, extended device lifecycles, and enforceable global regulations on e-waste management, digital health harms climate justice. Our ability to reframe the climate cost of digital health is also hampered by a lack of policies, frameworks and tools specific to reducing the carbon footprint and climate injustice in the context of digital health. So, what can we do to change this narrative at least from the Western world perspective?

## 4. Opportunities for Green Digital Health

Recognising that Earth’s systems are irrevocably changing due to excessive GHG emissions, the 2016 Paris Agreement, a legally binding international treaty, asks the world to hold “the increase in the global average temperature to well below 2°C above pre-industrial levels” and pursue efforts “to limit the temperature increase to 1.5°C above pre-industrial levels” [ 12 ]. To limit global warming to 1.5°C above pre-industrial levels, the world must reach net zero emissions by 2050 [ 23 ]. Recognizing the contribution of ICTs to global warming, the COP29 Declaration on Green Digital Action, signed by only 76 countries [ 53 ], attempts to address this issue. From the big polluters, only China, Russia, Japan and Brazil are signatories. From the digital health perspective, interventions are needed to implement the COP29 Declaration on Green Digital Action recommendations, including policy mechanisms that support sector-wide compliance. Digital health already plays a role in climate-resilient futures of planetary health, including during disaster responses [ 6 ], access to care through judicious design and use of telehealth [ 54 ], early warning systems and initiatives around One Health [ 55 ], and development of standardised terminologies to connect climate-related human health data [ 56 , 57 ]. Frameworks exist on how digital health can support GHG and waste mitigation action [ 58 , 59 ]. However, all these efforts are undermined by the fact that these digital health applications are simultaneously harming the planet, as discussed above. Our mission is to consciously and proactively develop green digital health. Below are many actions that we take starting today.

### 4.1 Advocacy for Greener Digital Health

We all have a role in advocating for best practices that balance patient care and environmental sustainability. This includes supporting the transition to renewable energy, using optimised energy-efficient cloud computing and environmentally sustainable telehealth solutions, supporting research into low-energy digital solutions, and encouraging the adoption of resource-efficient computing solutions that uphold climate justice [ 7 ]. Responsible data storage practices should be prioritised, such as deleting obsolete and redundant data, prioritizing lower-energy video protocols and implementing energy-efficient storage solutions. Additionally, advocating for low-energy blockchain alternatives and integrating environmental sustainability into discussions on big data, AI, and machine learning is essential [ 53 ].

We must advocate for the longer lifecycle of medical devices and wearables, recyclable materials, and manufacturer-led take-back programs that reduce e-waste. Sustainable procurement practices, including choosing suppliers committed to net-zero goals, reducing packaging waste, and adopting circular economy models, can support the healthcare supply chain to be environmentally responsible. Advocacy should also extend to integrating environmental sustainability policies in digital health governance. This includes developing green standards and certifications for digital health systems and embedding sustainability metrics into institutional digital transformation strategies. With knowledge and evidence already available, we have the tools to champion environmentally sustainable digital health solutions.

### 4.2. Environmentally Conscious Digital Health Curriculum

Educators are responsible for embedding environmental sustainability in digital health and informatics curricula. This starts with building carbon literacy and helping students understand the environmental impact of digital health technologies through principles of planetary health [ 70 ]. Teaching efficient coding and algorithm optimization reduces unnecessary computational loads, while simple habits such as reducing email overload and avoiding unnecessary file duplication can lower digital footprints at professional and individual levels ( Figure 4 ) [ 60 ]. When teaching the design of low-carbon digital health applications and knowledge dissemination platforms, the systems thinking approach is essential. This involves considering the digital health application system boundaries, including consumers, the planet, supply chains, mobile health apps, Internet of Things devices, telehealth equipment, and data storage [ 7 ]. Encouraging responsible video streaming, such as using compressed file formats and lower resolutions when high definition is unnecessary, and simplifying screen complexity is both environmentally sustainable and cost effective as it reduces energy consumption bills. Students must be taught how to advocate for sustainable production and recycling, aligning with climate justice principles [ 51 ]. By equipping our students, with this knowledge and skill set, we prepare future digital health practitioners to face the climate-related challenges of today and tomorrow.

**Figure 4.**
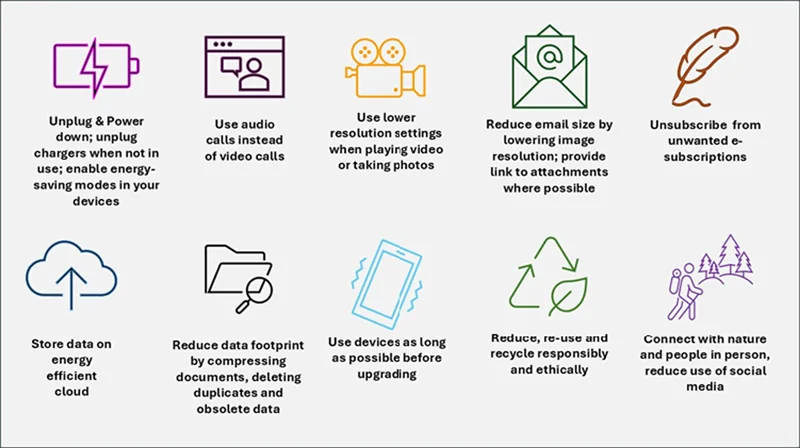
Reducing personal digital carbon footprint (based on [ 53 , 60 ]).

### 4.3 Digital Minimalism

“Digital minimalism” is a philosophy that promotes intentional and mindful technology use, focusing on what adds value while minimizing distractions and digital clutter [ 61 ]. In digital health, this philosophy could guide intervention design to minimise digital overload while ensuring high-quality patient care, safe decision-making, and effective digital health education. This means reducing redundant apps and platforms, integrating only essential systems into electronic health record (EHR)/electronic medical record (EMR) environment and adopting lean data management, i.e. storing only necessary and actionable patient data to reduce energy-intensive storage demands. It also includes implementing policies to support automated deletion of outdated or duplicate health records to optimize low-impact cloud storage. It may also mean implementing low-bandwidth telehealth solutions to ensure efficient, high-quality remote care without unnecessary data consumption. By adopting digital minimalism, there is also a reduction of e-waste in the system.

### 4.4 Greener Models of Care

Our existing models of care (care pathways) rest on clinical decisions about which services can be offered and how these services can be delivered. However, many care pathways are not value-based, prompting concerted efforts to reduce wasteful healthcare [ 44 , 62 ]. Transforming existing models of care towards value-based models is also suggested to mitigate healthcare’s carbon footprint and achieving net zero targets [ 62 ]. Greener care pathways balance environmental sustainability with high-quality, patient-centred healthcare through four principles: (i) disease prevention and health promotion; (ii) self-care and patient empowerment; (iii) lean service delivery; and (iv) lower carbon alternatives [ 63 ]. These same principles can also guide environmentally sustainable digital health solutions. For example, digital health can reduce unnecessary healthcare utilisation through decision support systems in EMRs, such as by minimising redundant pathology tests (also carbon-reducing activity) [ 64 ], promoting preventive care and early interventions to reduce avoidable hospital admissions [ 65 ] and limiting the use of environmentally harmful anaesthetic gasses [ 66 ]. Decision support systems can also reduce overprescribing and prioritise low-carbon pharmaceuticals, such as environmentally friendly asthma inhalers, where appropriate [ 67 ]. Furthermore, informatics methods such as process mining [ 68 ] could be applied to identify and re-design carbon-intensive pathways. All these actions fall within the scope of digital health practitioners, positioning them at the coalface of sustainable healthcare transformation.

### 4.5 Collaborate for the Environment at Local, National and International Levels

Climate change affects us all regardless of where we live. In addition to personal awareness, the business community increasingly recognises climate change’s impacts on economy, planetary and human health. Climate action is then not only viewed as necessary but also a valuable business opportunity [ 69 ]. However, time is running out to keep global warming temperatures below 1.5°C. This means that the digital health community/sector, for the benefit of planetary health and the well-being of future generations, needs to evolve its modus operandi, moving away from the traditional approach to research and implementation towards open access collaboration with local and global partners committed to net zero transition. Open-access resources allow us to freely share evidence-based practices on how digital health contributes to environmentally sustainable healthcare net zero agenda. This includes mentoring and supporting global efforts to embed sustainable digital health solutions as health is universal. Collaborations already exist, such as informatics and climate interest groups within the American Medical Informatics Association and the International Medical Informatics Association, although access is restricted to members. Our final challenge is then to establish open, environmentally sustainable digital platforms that facilitate knowledge exchange, joint research initiatives, and cross-industry partnerships that support planetary health. These platforms must be inclusive, ensuring that sustainable digital solutions are scalable and accessible, particularly in low-resource settings, driving meaningful acclimate action while advancing global health equity.

## 5. Conclusion

The digital health sector stands at a pivotal moment, where the choices we make today will shape not only the future of healthcare but also the health of the planet and future generations. While digital technologies have undeniable environmental costs, they also present an opportunity to drive environmentally sustainable innovation. By integrating green practices, such as energy-efficient data management and sustainable ICT device design, we can reduce technology’s environmental footprint while advancing global and planetary health outcomes. Achieving this meaningful change requires a collaborative approach at all levels of the healthcare system, involving healthcare providers, policymakers, industry leaders, and consumers. Through collaboration and accountability, digital health can fulfil its potential to improve lives while contributing to a healthier planet. Ultimately, the responsibility lies with all of us to include environmental sustainability and climate justice into digital health. Only by doing so can we build digital health systems that serve future generations, leaving a legacy of health and environmental well-being for both the planet and people.
